# Evaluation of Reference Centers for Special Immunobiologicals implementation

**DOI:** 10.1590/S1518-8787.2016050006183

**Published:** 2016-08-26

**Authors:** Laura Andrade Lagôa Nóbrega, Hillegonda Maria Dutilh Novaes, Ana Marli Christovam Sartori

**Affiliations:** I Departamento de Moléstias Infecciosas e Parasitárias. Faculdade de Medicina. Universidade de São Paulo. São Paulo, SP, Brasil; II Departamento de Medicina Preventiva. Faculdade Medicina. Universidade de São Paulo. São Paulo, SP, Brasil

**Keywords:** Immunization, Health Centers, Immunization Programs, organization & administration, Vaccines, supply & distribution, Program Evaluation, Health Services Evaluation

## Abstract

**OBJECTIVE:**

To describe the Reference Centers for Special Immunobiologicals and evaluate their implementation considering formal regulations.

**METHODS:**

We conducted a program evaluation, of evaluative research type. From August 2011 to January 2012, a questionnaire was applied to the 42 Reference Centers for Special Immunobiologicals existing in the Country, approaching the structure, human resources, and developed activities dimensions. We conducted a descriptive analysis of data and used a clustering for binary data with the squared Euclidean distance, by the farthest neighbor method, to aggregate services with similar features.

**RESULTS:**

We observed great diversity among the services in the three dimensions. The clustering resulted in five service profiles, named according to their characteristics. 1) Best structure: 12 Reference Centers for Special Immunobiologicals with the highest proportion of services with the minimum of rooms recommended, purpose-built vaccine refrigerators, preventive maintenance of the cold chain, and oxygen source. 2) Immunobiologicals distributor: six Reference Centers for Special Immunobiologicals that distributed more than applied immunogens; no doctor present for more than half of the working hours and no purpose-built vaccine refrigerators . 3) Incipient implementation: five Reference Centers for Special Immunobiologicals with inadequate structure, such as absence of purpose-built vaccine refrigerators, preventive maintenance of the cold chain and oxygen source; none had computer. 4) Vaccination rooms: 13 Reference Centers for Special Immunobiologicals, everyone did routine immunization, most participated in vaccination campaigns. 5) Teaching and research: six services, all inserted into teaching hospitals, developed researches and received trainees; most had doctors in more than half of the working hours.

**CONCLUSIONS:**

The evaluation of the Reference Centers for Special Immunobiologicals implementation was based on the profiles found and considered the official regulations: services categorized as “better structure” and “teaching and research” were considered implemented; “immunobiologicals distributor” and “vaccination room” services, partially implemented, and the ones with the “incipient implementation” profile, not implemented. The results of this evaluation can contribute to the reformulation of the services, considering the current context.

## RESUMO

**OBJETIVO:**

Descrever os Centros de Referência para Imunobiológicos Especiais e avaliar sua implantação considerando as regulamentações formais.

**MÉTODOS:**

Foi realizada uma avaliação de programa, tipo pesquisa avaliativa. De agosto de 2011 a janeiro de 2012, foi aplicado questionário aos responsáveis pelos 42 Centros de Referência para Imunobiológicos Especiais existentes no País, abordando as dimensões estrutura, recursos humanos e atividades desenvolvidas. Foi feita análise descritiva dos dados e utilizado agrupamento para dados binários com uso da distância euclidiana quadrática, pelo método do vizinho mais distante, para agregar serviços com características semelhantes.

**RESULTADOS:**

Observou-se grande diversidade entre os serviços nas três dimensões. O agrupamento resultou em cinco perfis de serviços, denominados de acordo com suas características. 1) Melhor estrutura: 12 Centros de Referência para Imunobiológicos Especiais com a maior proporção de serviços com o mínimo de salas preconizado, câmaras de vacinas, manutenção preventiva da rede de frio e fonte de oxigênio. 2) Dispensador de imunobiológicos: seis Centros de Referência para Imunobiológicos Especiais que mais dispensavam do que aplicavam imunógenos; sem médico presente por mais da metade do expediente do serviço e sem câmara de vacinas. 3) Implantação incipiente: cinco Centros de Referência para Imunobiológicos Especiais com estrutura inadequada, como ausência de câmaras de vacinas, de manutenção preventiva da rede de frio e de fonte de oxigênio; nenhum possuía computador. 4) Sala de vacinas: 13 Centros de Referência para Imunobiológicos Especiais, todos faziam imunização de rotina, a maioria participava de campanhas de vacinação. 5) Ensino e pesquisa: seis serviços, todos inseridos em hospitais de ensino, desenvolviam pesquisas e recebiam estagiários; a maioria possuía médicos em mais da metade do expediente.

**CONCLUSÕES:**

A avaliação de implantação dos Centros de Referência para Imunobiológicos Especiais baseou-se nos perfis encontrados e considerou as regulamentações oficiais: os serviços categorizados como “melhor estrutura” e “ensino e pesquisa” foram considerados implantados; os serviços “dispensador de imunobiológicos” e “sala de vacinas”, parcialmente implantados e os do perfil “implantação incipiente”, não implantados. Os resultados dessa avaliação podem contribuir para a reformulação dos serviços, considerando o contexto atual.

## INTRODUCTION

Patients with chronic diseases, such as immune deficiencies (congenital or acquired), neurological, hematological, and metabolic disorders, heart diseases, lung diseases, and others, or with exposure to risk situations, have a higher risk of infection or severe illness by certain pathogens and have recommendations for specific immunizations^15^ ^,^ ^24^ ^,^ ^25^. Several countries, such as United Kingdom, France, Germany, United States, Mexico, and Argentina, have established vaccination calendars for these individuals^1^ ^,^ ^2^ ^,^ ^4^ ^,^ ^7^ ^,^ ^8^ ^,^ ^13^.

To meet these special groups in Brazil, the *Programa Nacional de Imunizações* (PNI – National Immunization Program) created the Reference Centers for Special Immunobiologicals (CRIE), which are public and free vaccination units. These Centers provide vaccines and immunoglobulins not available on the PNI routine for individuals with a higher risk of infection or severe illness, and for those with contraindication of immunobiologicals used routinely. Besides, the CRIE are also responsible for the investigation and follow-up of cases of adverse events following immunization (AEFI)^15^. The first services were created in 1993. Until 2000, 34 CRIE were created and, since 2002, each Brazilian state has at least one of these services^16^.

Several publications discuss indications of special immunobiologicals or describe the care to specific groups in the CRIE^3^ ^,^ ^5^ ^,^ ^6^ ^,^ ^21^ ^,^ ^24^ ^,^ ^26^, but we did not find studies that evaluate the implementation of CRIE in national perspective.

The aim of this study was to describe the CRIE existing in Brazil, in 2011, and evaluate if their implementation occurred in accordance with the guidelines and regulations established by PNI.

## METHODS

The methodology adopted in the study was evaluation of health program of evaluative research type^19^. All managers of the 42 CRIE in the Country were invited to participate, identified by a list provided by the PNI General Coordination in June 2011. In 21 states, only one CRIE was identified, located in the capital. Six states and the Federal District had more than one service: Para (two), Bahia (two), Federal District (four), Sao Paulo (seven), Rio de Janeiro (three), and Rio Grande do Sul (three).

The CRIE are under the coordination of three instances: Brazilian Ministry of Health, by the PNI; State Secretariats of Health (SES); and the institution in which they are located (local level, usually higher complexity hospitals). The functioning and operation of CRIEs follow the Ordinance 48, from July 28, 2004^a^, which sets the minimum structure and the necessary human resources, also considering possible emergency care, since the target audience includes individuals with increased risk of presenting adverse events following immunization. The operation must occur on a time that allows the application of immunobiologicals (or their distribution to application in other service) in cases of urgency, as prophylaxis after exposure. The indications of immunobiologicals follow the PNI recommendations, listed in the CRIE Manual, revised and updated periodically by a group of experts^15^.

The data source was a semi-structured questionnaire developed for this study, consisting of 170 questions. The following program dimensions were studied: structure (existence of minimal physical area, institution in which it is inserted, equipment and inputs), human resources (number and training of professionals, workload, training for emergency care), and developed activities (application of immunobiologicals and their distribution to be applied in another service, assistance for adverse events following immunization, teaching and research activities). The regulations of the Ordinance that establishes general guidelines for CRIE operation guided the formulation of the questions, and the questionnaire was reviewed by the Technical Advisory of the General Coordination of PNI and by the Technical Management of Support to CRIE Management from the General Coordination of PNI.

The questionnaire was available in online platform (virtual environment), from August 2011 to January 2012, and answered by the managers of the CRIE, or their substitutes, after agreement by signing the informed consent form. The project was approved by the Research Ethics Committee of the Hospital das Clinicas of Faculdade de Medicina of Universidade de São Paulo (Protocol of research 0281/10).

In the descriptive analysis, we estimated the simple frequencies of the answers for the characterization of the services. To create service profiles with similar characteristics, we selected nuclear issues in the three evaluated dimensions and performed the analysis using the clustering for binary data with the squared Euclidean distance, by the furthest neighbor method (complete linkage). This technique allows one to move successively in the algorithm by clustering small groups in larger ones, according to the values of the squared Euclidean distance, producing a tree of groups named dendrogram. The cutoff level in the dendrogram is defined by the researcher and must represent the most suitable number of groups, in accordance with the research goals^9^.

## RESULTS

All 42 CRIE filled the questionnaire, with varying completeness and quality.

**Figure 1:**
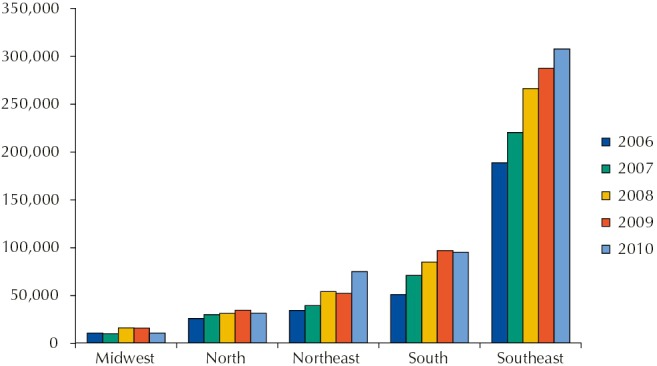
Number of immunobiologicals doses applied in the Reference Centers for Special Immunobiologicals (CRIE), by region and year, in the period from 2006 to 2010.

Figure 1 presents the number of immunobiologicals doses applied by the CRIE, from 2006 to 2010, by region and year (nine CRIE without information). In 2010, the CRIE accounted for 518,964 immunobiologicals doses applied. The record was made by applied doses and each person could receive more than one immunobiological. Few services (21.0%) recorded the attended cases in which the required immunobiological was not released. The total number of applied doses increased 66.0% in the studied period; but this increase was not uniform, being higher in the Northeast (118.0%), followed by South (87.0%), and Southeast (63.0%). In the North and Midwest there was not increase in the number of applied doses; however, this datum was not informed by 10 (59.0%) services of these regions. Thirty-five CRIE (83.0%) mentioned applying immunobiologicals of the PNI routine calendar and 31 (74.0%) reported participating in vaccination campaigns.

CRIE without information: 9

The 40 CRIE (95.0%) reported that they rendered assistance to adverse events following events; 36 (86.0%) mentioned counting on support of experts for this service; 34 (81.0%), with hospital support; and 28 (67.0%), with laboratory support (Table).

**Table:** Structural and human resources characteristics and developed activities in the Reference Centers for Special Immunobiologicals (CRIE) in accordance with the services and distribution profiles (%) of the structural and human resources characteristics and developed activities. Brazil, 2011.

Characteristics	Profile					

	G1	G2	G3	G4	G5	Total

	(n = 12)	(n = 6)	(n = 5)	(n = 13)	(n = 6)	(N = 42)

	%	%	%	%	%	%
Structure						
Proportion of services that are inserted into teaching institution^a^	58.0	0	0	0	100	31.0
Proportion of services that have minimal physical structure, according to the recommendation (reception, doctor’s office, preparation and/or vaccination room)^a^	92.0	50.0	60.0	54.0	83.0	69.0
Proportion of services that are inserted into building where other services operate^a^	75.0	100	80.0	92.0	100	88.1
Proportion of services that share the working area with other activities^a^	17.0	17.0	100	62.0	50.0	45.2
Proportion of services that have purpose-built vaccine refrigerator^a^	58.0	0	20.0	54.0	50.0	42.9
Proportion of services with preventive maintenance for cold chain^a^	92.0	83.0	20.0	0	83.0	52.4
Proportion of services that have electrical power generator^b^	91.7	50.0	80.0	46.2	100	71.4
Proportion of services that have oxygen source^a^	92.0	33.0	20.0	69.0	17.0	57.1
Proportion of services that have computer^a^	75.0	100	0	100	100	80.1
Proportion of services that have fax^a^	42.0	33.0	0	69.0	83.0	50.0
Human Resources						
Proportion of services that have doctor in more than 50.0% of working hours^a^	66.7	0	60.0	23.1	83.3	45.2
Proportion of services that have human resources training to meet emergencies^a^	100.0	17.0	20.0	69.0	67.0	64.3
Developed activities						
Proportion of services that apply routine immunobiologicals^a^	92.0	33.0	60.0	100	100	83.3
Proportion of services that apply more than 80.0% of the released immunobiologicals in their own CRIE^a^	50.0	33.3	60.0	92.3	83.3	66.7
Proportion of services that participated in vaccination campaigns in the last 5 years^a^	67.0	83.0	80.0	69.0	83.0	73.8
Proportion of services that meet non-presential solicitations (only the requests)^a^	100	67.0	60.0	85.0	83.0	83.3
Proportion of services that provide a counter-reference document to the patient/requester, when there is no indication of immunobiological^a^	100	67.0	40.0	54.0	67.0	69.0
Proportion of services that register the cases seen to which the immunobiological was not released^b^	16.7	16.7	20.0	23.1	33.3	21.4
Proportion of services with operation to the public more than 40 hours a week^a^	58.3	66.7	40.0	84.6	50.0	59.5
Proportion of services that have available telephone contact for 24 hours^a^	67.0	83.0	60.0	46.0	67.0	61.9
Proportion of services that meet people who received immunobiologicals in other health units and present adverse events following immunization^a^	83.0	83.0	80.0	92.0	100	88.1
Proportion of services that have support of experts for cases of adverse events following immunization^b^	91.7	100	80.0	76.9	83.3	85.7
Proportion of services that have laboratory support for cases of adverse events following immunization^b^	83.3	83.3	40.0	61.5	50.0	66.7
Proportion of services that have hospital support for cases of adverse events following immunization^b^	91.7	100	80.0	76.9	50.0	81.0
Proportion of services that rely on technical support Group for the discussion of cases of adverse events following immunization^b^	58.3	0	40.0	53.8	66.7	47.6
Proportion of services that receive trainees^a^	75.0	33.0	60.0	85.0	100	73.8
Proportion of services that do or did researches in immunizations^a^	42.0	0	0	15.0	100	31.0

G1: best structure; G2: immunobiologicals distributor; G3: incipient implementation; G4: vaccination room; G5: teaching and research

^a^ Questions used in the statistical clustering for the creation of CRIE profiles.

^b^ Characteristics not included in the creation of the profiles.

Nine services reported having no doctor, at the time of the research, and four reported lack of nurse. There were trained professionals for emergency care in 27 services (64.0%).

Regarding the infrastructure, lack of sufficient cold chain equipment was reported by 29 CRIE managers (69.0%) and 35 (83.0%) reported use of domestic refrigerators. Source of oxygen was available in 24 CRIE (57.0%).

**Figure 2:**
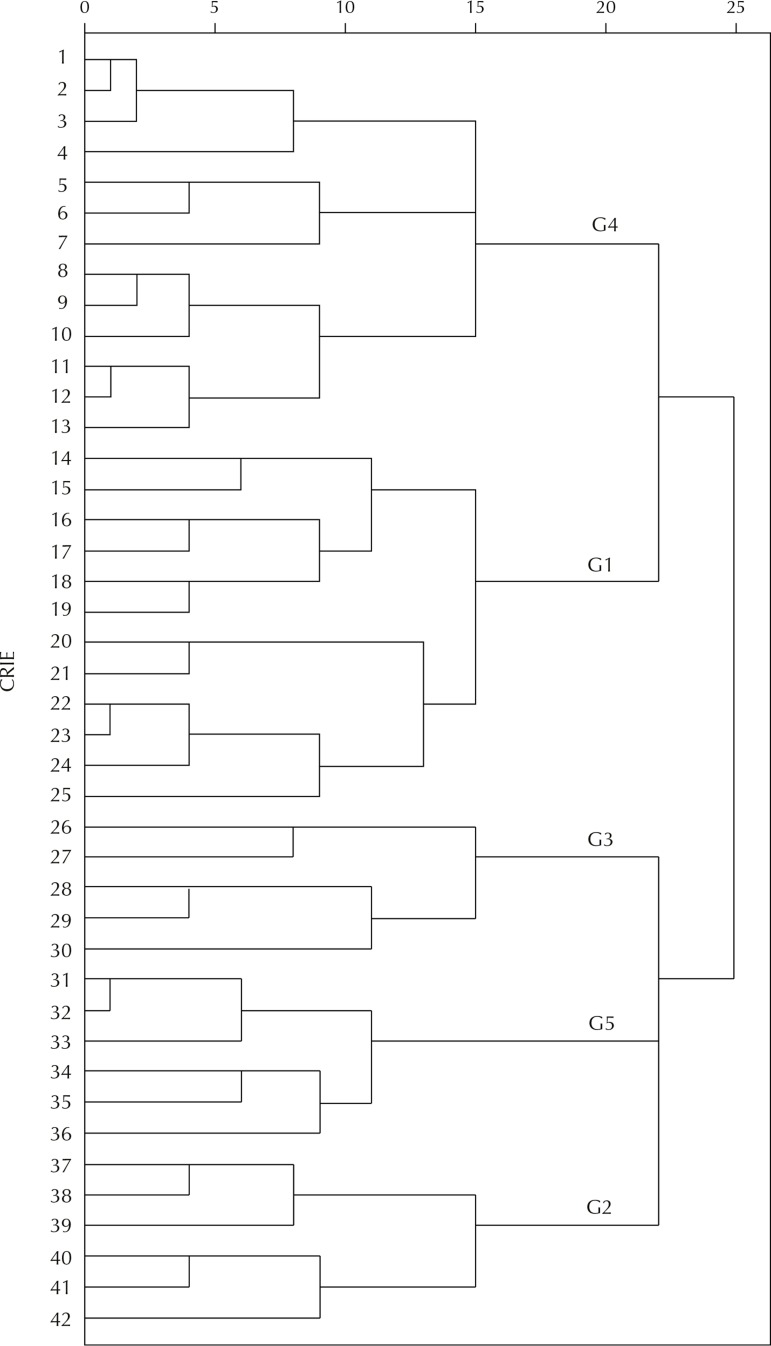
Dendrogram of the clustering for binary data with the squared Euclidean distance, by the farthest neighbor method, with formation of five groups of services with similar features.

CRIE: Reference Centers for Special Immunobiologicals; G1: best structure; G2: immunobiologicals distributor; G3: incipient implementation; G4: vaccination room; G5: teaching and research

In the dendrogram analysis resulting from the application of the grouping method for binary data, using the Euclidean distance squared, by the furthest neighbor method (complete linkage), to the selected data for the characterization of the services, we opted by cutting the squared Euclidean distance measure in 20 points. Five clusterings resulted from this, in which the services were considered to have relevant characteristics in common and that differed them from other clusterings (Figure 2).

Group 1 was named “best structure” profile, because it featured the largest number of services that had the minimum recommended rooms (reception, doctor’s office, and preparation or vaccination room). This group also showed greater proportion of services with presence of oxygen source, purpose-built vaccine refrigerators, and preventive maintenance of the cold chain. All services of this group had trained professionals to meet emergencies. Most received trainees, but less than half did research activities (Table).

Group 2 was named “immunobiologicals distributor” profile, for being the group with services that applied fewer doses of immunobiologicals in relation to the total distributed immunogens (administered and distributed) by the service. It also featured the lowest frequency of services that applied the vaccines of the routine calendars. Only half of the services had minimal physical structure and none of them had purpose-built vaccine refrigerators. It was the only group in which no CRIE had doctor present during more than half of the working hours. This group also showed the lowest frequency of trained professionals to meet emergencies.

Group 3 was named “incipient implementation” because it includes services with insufficient structure, according to the foreseen in the Ordinance that regulates the CRIE. None of them had unique physical area, few had purpose-built vaccine refrigerator and oxygen supply and the services had the lowest rate of preventive maintenance of the cold chain. None of these CRIE had computer or fax. Less than half worked more than 40 hours a week, considered as minimum necessary period for application and distribution of immunobiologicals satisfactorily in the CRIE.

Group 4, named “vaccination room” profile, included services that showed features that were more similar to a conventional vaccination room. All services did routine vaccination, and most of them applied the majority of their immunobiologicals and participated in vaccination campaigns. Most services worked more than 40 hours a week.

In Group 5, called “education and research” profile, all CRIE were inserted into teaching hospitals, developed research activities and received trainees. This group presented the best index of presence of doctors. Most services had appropriate minimum physical structure. All applied routine vaccines and most applied more than 80.0% of released immunobiologicals and participated in vaccination campaigns.

**Figure 3:**
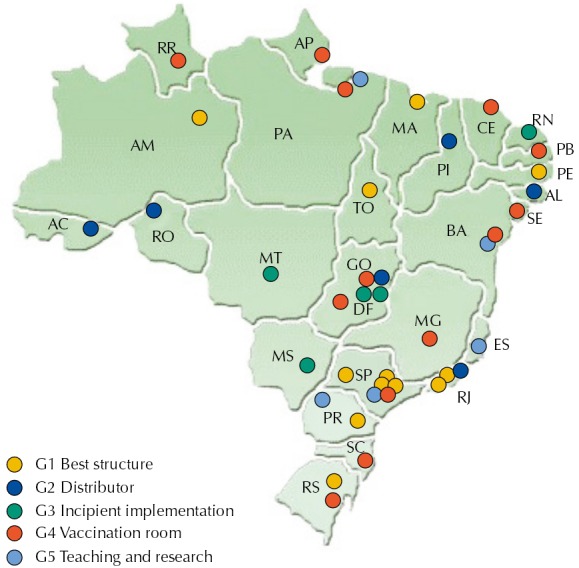
Geographic distribution and profile (G1 to G5) of the Reference Centers for Special Immunobiologicals in Brazil by state, in 2011.

Twelve of the 18 CRIE of the “better structure” and “teaching and research” groups were located in the Southeast and South regions (Figure 3). From the five services of the “incipient implementation” profile, four were located in the Midwest region. In the North and Northeast, there is no evidence of the predominance of any group.

**Figure 4:**
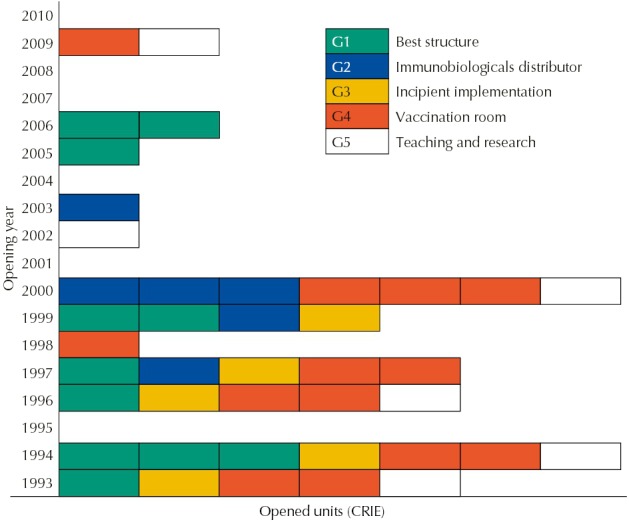
Distribution of Reference Centers for Special Immunobiologicals (CRIE) according to the profile (G1 to G5) and opening year.

All CRIE of the “incipient implementation” group were created before 2000, while, in the “better structure” and “teaching and research” groups, we observed services created at different times, from 1993 to 2009 (Figure 4).

The degree of CRIE implementation was evaluated considering the Ordinance that regulates the services. The CRIE with the profiles called “better structure” and “teaching and research” were considered implemented. The CRIE with the “immunobiologicals distributor” and “vaccination room” profiles were considered partially implemented. The CRIE of the “incipient implementation” profile were considered not implemented.

## DISCUSSION

This is the first systematic study that evaluated the implementation of CRIE in national perspective based on primary data, analyzing the structure, human resources, and developed activities dimensions. The data showed the diversity of the services’ situation nearly two decades after the creation of the special immunobiologicals program by PNI, indicating that the implementation of CRIE occurred without uniformity.

Regarding the CRIE distribution across the Country, we did not observe a population coverage criterion for the definition of the number of services by state. According to personal information of state managers of immunization, distribution of these immunogens to the cities via Health Regions seems to have been implemented in some states, as an alternative to increase patients access to special immunobiologicals.

We expected to observe a relation between the time of opening of the services and the degree of implementation, but the results do not prove this hypothesis, because all services of “incipient implementation” were inaugurated for over a decade (Figure 4). On the other hand, there seems to be a relation between the region where the CRIE were located and the degree of implementation, since most CRIE of G1 and G5 were located in the Southeast, while most CRIE of G3 were located in the Midwest region.

Some faced barriers for the implementation of CRIE may have been caused by changes in policies for the Brazilian Unified Health System (SUS) over time. The conventional vaccination rooms were decentralized with the municipalization of health, what did not occurred with the CRIE. The local context of the institutions that house the CRIE, regarding the available structure, human resources, and their priorities, appear to have been instrumental in the implementation and performance of the services.

Several factors may have contributed to the increase in the number of immunobiologicals doses applied by CRIE, such as: increased target audience, disclosure of the existence of CRIE and dissemination of knowledge about the recommendations of special immunobiologicals. This increase was most evident in the Northeast, followed by the South and Southeast regions. The absence of an increase in the number of doses applied in the North and Midwest regions may be due to information bias, since more than half of the services of these regions did not inform the number of immunobiologicals doses applied. Obstacles to the growth of some services may be arising from the inadequacy of the physical area, lack of cold chain equipment (or their inadequacy regarding regulations)^14^, and the lack of essential human resources. These factors can also impair the activities of evaluation and application of immunobiologicals, as well as the care of emergencies and investigation of adverse events following immunization. This panorama may have changed in recent years, since some improvements may have been implemented, such as the transfer of funds from the Ministry of Health to the states for adequacy of the cold chain^b^.

Despite the heterogeneity within the groups, the profiles called “better structure” (G1) and “teaching and research” (G5), which amounted to 18 services (43.0%), resembled more what was proposed in the Ordinance that regulates the CRIE, being considered implemented. The characteristics that distinguished these two groups from the others were the higher proportion of services with more complete structure (G1) and higher proportion of services that developed activities such as application of immunogens in the very CRIE, participation in campaigns, application of routine vaccinations, and teaching and research activities (G5).

The initial proposal for all CRIE was that they were privileged spaces for the development of teaching and research, training and capacity building. Some CRIE have developed studies in specific populations immunizations and adverse events following immunizaiton^3^ ^,^ ^6^ ^,^ ^10^ ^-^ ^12^ ^,^ ^17^ ^,^ ^18^ ^,^ ^20^ ^-^ ^23^. The CRIE that comprise the two most well-structured profiles could further develop teaching and research activities, counting on greater support and encouragement of the SES and the Ministry of Health/PNI. Meanwhile, placing the same requirements for services with very different conditions may hamper the identification of which activities could be considered more relevant to the different types of CRIE. Although not presenting appropriate conditions to develop research, the “immunobiological distributor” and “vaccination room” profiles could invest in training and capacity building for health professionals in their coverage area, as well as in the strengthening of their main activities of application and dispensing of special immunobiologicals. The services with “incipient implementation” profiles require extensive adequation of the structure and human resources and reorganization of the developed activities to find their vocation.

Regarding the limitations of this study, the results must be analyzed with caution, since they were based on a questionnaire applied at a distance. With this type of assessment tool and without on-the-spot inspection, the responses must be treated as mentioned information and are subjected to the subjectivities of the respondents, related to their personal opinions or interpretations of the questions. Some questions were unused because they are not well formulated or their formatting has raised questions in the online form. Besides, the choice of the questions for the statistical analysis may have reflected in the final outcome of the implementation evaluation.

Additionally, the introduction of new vaccines into the PNI routine immunization calendar, such as the 10-valent pneumococcal conjugate and meningococcal C conjugate (2010), IPV (2012), varicella (2013), and hepatitis A (2014), may have altered the demand for vaccines in the CRIE. Besides, new services were created (at least three more CRIE were opened since the end of data collection until 2014, in Minas Gerais, Rio de Janeiro, and Acre). However, there is no evidence that substantial changes have occurred in the services since the data collection.

Considering that less than half of the services were classified as fully implemented, the results of this study may contribute to the restructuring of the services, including the review of the role of CRIE and reformulation of the special immunobiologicals program, based on the current context.

## Footnotes

a Ministério da Saúde, Secretaria de Vigilância em Saúde. Portaria nº 48, de 28 de julho de 2004. Institui diretrizes gerais para funcionamento dos Centros de Referência para Imunobiológicos Especiais – CRIE, define as competências da Secretaria de Vigilância em Saúde, dos Estados, Distrito Federal e CRIE e dá outras providências. *Diario Oficial Uniao*. 29 jul 2004;seção 1:63.

b Ministério da Saúde, Secretaria de Vigilância em Saúde. Portaria nº 3.301, de 26 de dezembro de 2013. Autoriza o repasse financeiro de investimento do Fundo Nacional de Saúde aos Fundos de Saúde Estaduais e Municipais para aquisição de equipamentos, material permanente e/ou unidade(s) móvel(is) para fomento e aprimoramento das condições de funcionamento da Rede de Frio. *Diario Oficial Uniao*. 26 dez 2013;seção 1:256.
